# Can I dismiss the stereotype – as my teacher did? Influence of stereotype activation and an immigrant teacher on student learning

**DOI:** 10.1007/s11218-022-09707-5

**Published:** 2022-07-05

**Authors:** Karen Ollrogge, Madita Frühauf, Theresa Mros, Julia Böttger, Elisabeth Höhne, Nele McElvany, Lysann Zander, Bettina Hannover

**Affiliations:** 1 Department of Education and Psychology, Freie Universität , Berlin, Germany; 2 Leibniz Universität, Hannover, Germany; 3 Technische Universität, Dortmund, Germany

**Keywords:** Stereotype activation, Ingroup role model, Turkish immigrant students, Mitigation of stereotype threat, Vocabulary learning

## Abstract

Lower vocabulary in German is repeatedly reported for students with Turkish migration background attending school in Germany. We investigated whether in students of Turkish descent (a) learning vocabulary is impaired when the teacher activates the negative stereotype that students with Turkish family language learn less well and (b) whether a Turkish-origin teacher, as an ingroup expert model, can mitigate negative effects of the activation of the stereotype. In an experimental study, Turkish- and German-origin students (*N* = 182) living in Germany worked individually on a tablet on a vocabulary learning task instructed by a teacher in a video tutorial who introduced herself with either a Turkish or German name. Before the task, the teacher either mentioned that students in general (no stereotype activation) or students who speak Turkish in their families (stereotype activation) often have difficulties acquiring new vocabulary. A multiple-group regression analysis showed that Turkish-origin students learned significantly more under stereotype activation with the Turkish-origin teacher than in all other conditions. Results suggest that students are particularly motivated to learn when the teacher represents their ingroup targeted by negative stereotypes and openly addresses potential difficulties students of the stigmatized ingroup may encounter. We discuss the findings in light of the literature on stereotype threat and on the role of ingroup expert models.

## Introduction

As the latest Programme for International Student Assessment (PISA) study from 2018 showed once again, in many European Union member states, students with a migration background lag behind their non-immigrant peers in skills in the language of instruction at school (European Commission, 2019). Within the European Union, Germany is the country with the widest gap in language skills between students from immigrant and non-immigrant families (European Commission, 2016; 2019).

In Germany, the largest subgroup of students with an immigrant background is of Turkish descent (Statistisches Bundesamt, 2021b). This subgroup is also the one that is considered particularly disadvantaged regarding German language skills (Relikowski et al., 2015). This is a matter of concern as a good command of the language of instruction in school is a filter variable for successful learning in all subjects (e.g., McElvany et al., 2017; Kempert et al., 2016), and thus, for educational participation and skill acquisition more generally (e.g., Aktionsrat Bildung, 2016; Autorengruppe Bildungsberichterstattung, 2016).

A central aspect of language competence is vocabulary, the totality of words in the mental lexicon. Students of Turkish descent have been found to have quantitatively lower vocabulary in German, even if they have spent their entire, or a significant part, of their educational biography in Germany (e.g., Relikowski et al., 2015; Schwippert & Habben, 2013). To foreshadow the aims of our study, first, we wanted to find out whether students of Turkish descent are impaired in their learning of new German vocabulary by the activation of a negative stereotype related to their Turkish family background, and second, we wanted to examine whether negative effects of the activation of a stereotype can be mitigated by a teacher of Turkish origin who is a successful ingroup model for students of Turkish descent.

### Implications of negative stereotypes about Turkish-origin students

Stereotypes are beliefs about what characteristics (e.g., traits, interests, abilities, behaviors) members of particular social groups (e.g., ethnic groups) have or should have (e.g., Hamilton & Uhles, 2000). If a stereotype includes negative assumptions regarding a group’s academic abilities, individuals belonging to that group can be impaired in their capability to perform academically once the stereotype is activated: the concern about possibly confirming the stereotype oneself draws on mental and psychological resources, causing the person to perform below his or her potential, ultimately resulting in a confirmation of the negative stereotype. This phenomenon is called stereotype threat (Steele, 1997; Steele & Aronson, 1995; for meta-analyses on the effects of stereotype threat see Appel et al., 2015; Nguyen & Ryan, 2008). Significant mediating mechanisms have been identified as including physiological arousal, anxiety, avoidance motivation, altered cognitive processes (e.g., reduced working memory capacity, impaired memory recall, impaired executive functions, task-irrelevant cognitions), and learning-relevant behaviors (e.g., effort expenditure; for reviews see Pennington et al., 2016; Spencer et al., 2016).

Students of Turkish descent living in Germany face negative stereotypes about their academic competence. They are affected by negative expectations their peers (Latsch & Hannover, 2014) and teachers (Lorenz, 2021) at school have about children and adolescents with an immigrant background. In addition, they face specific negative stereotypes about Turkish immigrants as a group. Kleen et al. (2019) found that while preservice teachers of Turkish descent held positive implicit attitudes toward Turkish-origin students, negative implicit attitudes prevailed in German-origin preservice teachers. Froehlich and Schulte (2019) report that university students in Germany thought that most people would think of Turks as only moderately competent and as less competent than other immigrant groups, such as Italians, Russians, and Poles.

Consistent with these findings, other studies show that immigrant students in Germany are prone to become victims of stereotype threat. For instance, Sander et al. (2018) found that children with a migration background attending elementary school in Germany acquired fewer German words in a vocabulary learning task when being told that children who speak a language other than German at home often have difficulties learning new words. For immigrant students of Turkish descent attending school in Germany, the following studies reported performance impairments due to stereotype threat: Martiny et al. (2014) found that this group of students underperformed in a mathematics test after being told that German- and Turkish-born individuals differed in their mathematics competence. Froehlich et al. (2016) found that when a verbal ability test was described as being diagnostic of verbal intelligence, Turkish-origin students showed lower performances to the extent that they endorsed an entity theory of intelligence. Mok et al. (2017) found that only when students had to indicate their Turkish background directly before taking a verbal ability test, did they underperform to the extent that they endorsed vertical collectivism (i.e., the perception of one’ s self as part of a collective and the acceptance of inequalities within that collective). With this background in mind, we expected to see stereotype threat effects in our study for the stigmatized group of Turkish-origin students.

As substantiated in a recent meta-analysis of 33 experiments by Appel and Weber (2021), stereotype threat effects are only observed among members of the respective stigmatized group, while among nonmembers the activation of a negative stereotype has either no effect or even a performance-enhancing effect. Walton and Cohen (2003) coined the term stereotype lift for such performance-enhancing effects. They suggest that stereotype lift effects, i.e., a boost in performance caused by the activation of a negative outgroup stereotype, result from downward social comparisons with the stigmatized outgroup. Such comparisons strengthen non-stigmatized individuals’ self-efficacy and self-worth as well as their expectation to be treated with respect and of not being assigned the low status of the outgroup even if they were to fail. Consistent with the view that stereotype lift effects are due to downward social comparison processes, Mendoza-Denton et al. (2008) found that Asians and men, who are favorably stereotyped regarding mathematics, performed best when assured that the respective outgroup’s disadvantage was stable and immutable; i.e., in an experimental condition in which they were primed with (a) the information that their ingroup outperformed the outgroup in mathematics and (b) an entity view of ability. In their meta-analysis, Appel and Weber (2021) conducted separate analyses on those studies which included a control group of non-stereotyped individuals (*k* = 12) and found a mean effect size of *d* = 0.17 for stereotype lift effects. Against this background, we expected to see stereotype lift effects in our study for students of German descent.

### Stereotype threat effects on learning

Research on stereotype threat has typically investigated its effect on performance, i.e., individuals were observed in a testing situation in which they had to recall previously acquired knowledge or apply previously acquired competencies (e.g., Appel & Kronberger, 2012; Froehlich et al., 2018; Pennington et al., 2016). For instance, Froehlich et al. (2016) and Mok et al. (2017) had students work on a reading comprehension task from the PISA test. Martiny et al. (2014) asked their participants to solve problems from a nonverbal intelligence test and from the mathematical PISA test. The results of these studies suggest that the activation of a negative stereotype impairs participants’ capability to access knowledge from long-term memory or their efficiency in applying problem-solving skills and procedures. However, it is possible that stereotype threat not only hampers stereotyped individuals’ capability to demonstrate their abilities, but also their capability to learn and acquire new skills and knowledge. Whether processes involved in learning are impaired by negative stereotypes in a similar way has been rarely studied (Rydell & Boucher, 2017). We think doing so is important, as it is likely that the lower performance level of Turkish-origin students is not only due to stereotype threat in testing situations, but also due to negative stereotypes affecting the learning process itself.

Some studies already point to negative effects of stereotype threat on learning. Rydell et al. (2010a) found that women under stereotype threat acquired less mathematical information and required more time to absorb relevant information. Participants in another study by Rydell et al. (2010b) underperformed in a perceptual learning task under stereotype threat. Appel et al. (2011) found that stereotype threat interfered with learning-relevant behaviors, such as note-taking activities. Taylor and Walton (2011) showed that students under stereotype threat learned fewer new words than students in a control condition. Boucher et al. (2012) separated learning and performance conditions: women in a stereotype threat condition showed poorer learning outcomes than women (a) in a control condition, (b) for whom stereotype threat had been removed prior to the learning situation, and (c) for whom stereotype threat had been removed after the learning phase but before the final test. In our study, we investigated the effects of stereotype threat in a learning rather than a testing situation to gain a better understanding of how to support knowledge gains and skills acquisition in negatively stereotyped students of Turkish descent.

### Mitigation of negative effects of stereotype activation by a Turkish-origin teacher

While in Germany about four in ten students (38.1%) at a general education school have an immigrant background (Statistisches Bundesamt, 2022; Table 1.1), this is true for only about one in ten teachers (13%; special analysis of data from the Microcensus 2020, provided at our request on October 15, 2021 by Statistisches Bundesamt, 2021a). Of the already small proportion of teachers with an immigrant background, only a small percentage is of Turkish origin^1^. These numbers imply that it is extremely rare for students of Turkish descent to encounter an ingroup member as a teacher. As a result, little use can be made of the resource that ingroup role models can be for stigmatized students.

Various theoretical approaches suggest that ingroup role models can mitigate the effects of negative stereotypes on stigmatized individuals, each postulating slightly different mediating processes. Steele et al. (2002) assume that an expert role model counters a negative stereotype by personally demonstrating to ingroup members the possibility of a successful educational trajectory despite shared barriers. Similarly, Marx et al. (2013) suggest that ingroup role models mitigate threat because they invalidate a negative stereotype by sending the (implicit) message that others also affected by the stereotype can succeed, too. Liu et al. (2021) argue that by simultaneously carrying stereotyped characteristics and succeeding, ingroup experts prevent stereotype threat by changing individuals’ beliefs about the stereotype, namely the belief that members of the stereotyped group are unable to succeed due to unchangeable, stable characteristics. Dasgupta (2011) proposes that contact with successful ingroup experts works like a “social vaccine”, i.e., it inoculates individuals against self-doubt in high-stakes environments. This is achieved by the student feeling a stronger sense of belonging, increased self-efficacy, more attraction to difficult challenges, and less threatened (cf. Dasgupta, 2011, p. 234). Chaney et al. (2018) argue that stigmatized individuals constantly calibrate the likelihood of being negatively stereotyped in the respective setting. In this situation, expert models who are affected by a similar stereotype convey that negative stereotyping is less likely, as they are believed to not endorse the negative stereotype.

What empirical evidence is there that ingroup experts do in fact mitigate the impact of negative stereotypes on stigmatized individuals? Consistent with Chaney et al.‘s (2018) assumption that ingroup role models are believed to not endorse the negative stereotype, Glock and Kleen (2019) found that ethnic minority teachers had more positive attitudes toward ethnic minority students compared to ethnic majority teachers. In several studies investigating female students in STEM (i.e., science, technology, engineering, and mathematics), Stout et al. (2011) showed that contact with advanced female peers, professionals, or professors promoted positive attitudes towards STEM, identification with STEM, greater self-efficacy as well as more effort on STEM tests. In a meta-analysis of 45 studies on the impact of ingroup role models on interest and performance of individuals from underrepresented groups in STEM, Lawner et al. (2019) found a positive albeit small effect (which was insignificant when considering only lab studies). In their meta-analysis of 26 experimental intervention studies (comprising 1,401 research participants) in which ingroup role models were supposed to mitigate impairments due to stereotype threat, Liu et al. (2021) found an effect size of *d* = 0.63, suggesting a moderately strong performance improvement. In our study, we wanted to test the assumption that a Turkish-origin teacher, leading students of Turkish descent through a novel learning task, would mitigate the negative effects of an activated stereotype about their group. We expected that Turkish-origin students’ learning gains would be reduced if a stereotype about their group was named by a German teacher, but not if the stereotype was mentioned by a Turkish teacher.

### Study overview and research hypotheses

Previous research suggests that students of Turkish descent attending school in Germany are threatened by negative competence-related stereotypes about their group. In our study, we examined German vocabulary as a key aspect of language competency. Complementing studies showing that students of Turkish descent in Germany underperform in testing situations when a negative stereotype about their group is being activated, we investigated whether stereotype threat also impairs their capability to acquire new knowledge and whether a teacher of Turkish descent can mitigate the threat. The stereotype activated in our study pertained to language competence as well: The teacher mentioned that students who – besides German – also speak Turkish at home often have difficulties acquiring new German vocabulary. As stereotype threat effects only occur in individuals who self-identify as members of the stigmatized group, we operationalized a Turkish background by asking students whether they ever speak Turkish at home. Students who indicated to exclusively speak German were assigned to the non-targeted group of German students.

For students with Turkish family language, we expected the following: ingroup expert models can mitigate the threat emanating from a negative stereotype as they are both a member of the stigmatized group and successful (e.g., Liu et al., 2021). Therefore, compared to the control condition, when exposed to a negative stereotype about students with Turkish family language, Turkish-origin students learn less but only when instructed by a German-origin teacher (Research hypothesis 1).

For students of German descent, we expected them to engage in downward social comparisons when the negative stereotype was activated. Research hypothesis 2 is thus: compared to the control condition, when exposed to a negative stereotype about students with Turkish-family language, German-origin students learn more. We further explored whether learning gains in students with German as the family language would differ according to teacher origin, with no directed hypothesis.

## Method

### Sample

Three hundred thirty-eight students participated in the study. Consent to participation was obtained from both students and their parents. Students attended 18 ninth or tenth grade classes from five high schools of upper (“Gymnasium”, from which students graduate with eligibility for university studies) or lower (“Integrierte Sekundarschule”, from which students leave with the eligibility for vocational education and training) academic track in a large city in Germany. We excluded the following participants: Students who completed the survey in an unrealistic time (− 2 *SD*) or had a statistically outlying learning growth (± 2 *SD*; *n* = 15), as well as students who did not indicate their type of school (*n* = 9), or their family language (*n* = 5). Of the remaining 309 participants, 91 spoke exclusively German, 91 exclusively Turkish or Turkish and German, and 127 a language other than Turkish or German in their family. Given the focus of our hypotheses, only students who either exclusively spoke German or Turkish (exclusively or together with German) in their family were included in all further analyses^2^. Our final dataset thus consisted of 182 students (91 with German and 91 with Turkish family language). The experimental procedure which was applied for the group of students who spoke a language other than Turkish or German in their family will not be described further below.

Of the 182 students included in our analyses, 53.3% were female, 42.9% were male, and 1.6% self-identified as diverse. When calculating students’ mean age, we excluded 14 students who had provided unrealistic information (students who said they were younger than 13 or older than 18 years of age). The mean age of the 175 students providing realistic age-related information was 14.77 years (*SD* = 0.77).

### Experimental design and procedure

The study was approved by the School Senator of Berlin and the Ethics Committee of Freie Universität Berlin. Students were tested in the setting of their classroom by a team of 2–3 test administrators during regular class hours. After a brief standardized instruction by the test administrators, students completed the survey independently on tablets. We used the software LimeSurvey (Version 3.28.0; LimeSurvey GmbH, 2015) to program the study and to randomly assign students to the experimental conditions.

**Figure 1:**
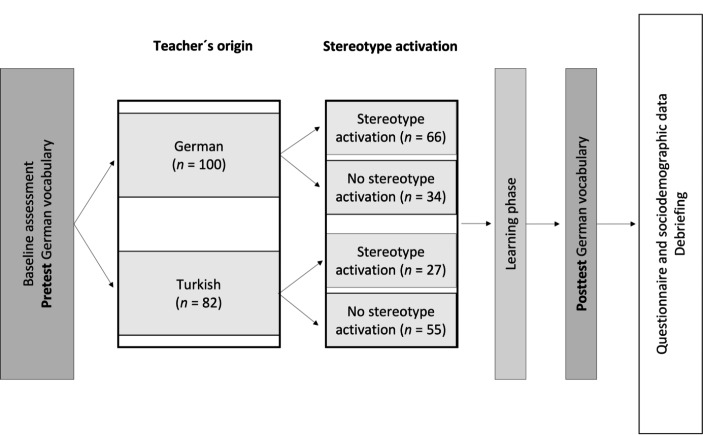
*Study Design*

In a pretest-posttest control group design, students were randomly assigned to one of four different experimental conditions resulting from the fully crossed combination of the two independent variables: teacher origin (German vs. Turkish) and stereotype activation (activation vs. no activation). When the student started the experiment on the tablet, LimeSurvey randomly assigned the adolescent to one of the four conditions: German-origin teacher with stereotype activation, Turkish- origin teacher with stereotype activation; German-origin teacher without stereotype activation, or Turkish-origin teacher without stereotype activation. Each student then watched a video on the tablet of a female teacher guiding through the study. Figure 1 shows the study procedure as programmed on the tablets. At the beginning of the video, the teacher explained that the study’s goal was to find out how students learn best, highlighting that it was about learning (and not performance). This instruction was followed by a vocabulary test (pretest) in which one word at a time was presented and the student had to select the appropriate synonym from a list of five words. Following the pretest, the experimental treatments were administered (see below for details). The student was then guided through the learning phase. Here, the words shown during the pretest were presented again, this time each accompanied by explanations of their semantic meaning. The following posttest contained the same words as the pretest. Each student finally received a debriefing explaining the study’s aim.

#### Manipulation of teacher origin

Following the pretest, the teacher introduced herself either with a typical Turkish name and background (“My name is Merve Yıldırım. I’m a teacher and live in Berlin. My parents are from Turkey. Before I was born, they moved to Berlin and we lived here ever since.“) or with a typical German name and background (“My name is Julia Schmidt. I’m a teacher and live in Berlin. My parents are from North Rhine-Westphalia. Before I was born, they moved to Berlin and we lived here ever since.“). The actress was a young German woman of Turkish origin. She spoke both languages fluently and her appearance could have been interpreted as both German and Turkish. To make the teacher’s origin more salient, she wrote her name on the blackboard in the background. Also, all further instructions were presented together with a small picture and the teacher’s name (Ms. Yıldırım/Ms. Schmidt).

#### Manipulation of stereotype activation

In the video, the manipulation of teacher origin was followed by the stereotype activation. In the stereotype activation condition, for Turkish- and German-origin students the teacher said that young people (“Jugendliche”, a gender neutral term) have difficulties in learning new German words when Turkish is also spoken at home. In the control condition (no stereotype activation), irrespective of students’ family language, the teacher said that young people often have difficulties learning new words.

### Instruments

#### Vocabulary pretest and posttest

The vocabulary test and the learning material were piloted in four ninth grade classes. Twenty-seven difficult German words were tested, from which we selected the 15 which were the least familiar to students, and for which students made the greatest learning gains. Two additional and particularly easy words (“icebreakers”) were presented at the beginning of the vocabulary test to allow an easy start. The student’s task was to choose a synonym for the word from a list of five words (e.g., target word: marode; word list: 1) modern, 2) broken, 3) moderate, 4) mixed, 5) colossal). Students’ answers in pretest and posttest were coded dichotomously (0 = not correct, 1 = correct). The icebreakers were excluded from the score, and thus, a maximum of 15 points could be obtained. Reliabilities were satisfactory for the pretest (α = .66) and posttest (α = .86). The learning score (ranging from 0 to 15 words) was obtained by subtracting the number of items solved correctly in the pretest from the number of items correctly solved in the posttest.

#### Learning material: Dictionary entries and closes

In the learning phase, the student was instructed to learn the 15 words presented in the pretest. The learning phase consisted of two parts. In the first part, each of the 15 words was explained using a fictitious dictionary entry consisting of an explanation of the word’s semantic meaning and an example sentence (e.g., target word: “ramshackle” - meaning: “run down, ruined” / example sentence: “The ramshackle building is in danger of collapse because it has not been maintained or inhabited for fifty years”). In the second part, the student was asked to complete sentences in each of which one word was missing. One of the 15 previously learned words had to be selected (from a list of eight) that completed the sentence in a meaningful way (e.g., “The entire basement was under water because the _____ condition of the pipes had caused water damage.“ Here, the word “ramshackle” had to be inserted). Afterwards, the student was given the correct answer so that she/he could see whether she/he was right or wrong.

#### Sociodemographic data

At the end of the survey, the student was asked to indicate their gender, age, and family language. Family language was operationalized via the following question (Mang et al., 2018): “Which language do you usually speak when you talk to the following people?” The student was asked to answer this question separately regarding “parents”, “siblings”, and “other relatives (e.g., grandparents, aunts, uncles)”. For each of the three groups of people, response options were: 1) only German, 2) only a language other than German, 3) both German and another language. Students who indicated to communicate with all three groups exclusively in German were categorized as students with German family language. Students who indicated that they usually speak a language other than German or both German and another language with any of the three groups of people were then further asked which language/s they had already learned as a young child (response options: German, Turkish, Arabic, Polish, Russian, another language). Students who indicated that they learned Turkish (exclusively or in combination with any other language/s) already as a child were categorized as students with Turkish family language.

### Statistical analyses

Statistical analyses were performed with Mplus version 8.7 (Muthén & Muthén, 2017) using a robust maximum likelihood (MLR) estimator. At first, descriptive statistics for Turkish- and German-origin students as well as for the overall sample were calculated. In addition, we tested mean differences and standardized mean differences of students’ learning gains between the different experimental conditions using simple linear regression analyses. In the next step, we regressed students’ vocabulary gain scores on our predictor variables: teacher origin (level 1: German = 0, Turkish = 1), stereotype activation (level: 1: no activation = 0, activation = 1), and the interaction between teacher origin and stereotype activation, while controlling for school type (level 2: upper academic track = 0, lower academic track = 1) in order to control for learning gains differing due to students’ academic track. As we were interested in differential effects between family language groups (students with German vs. Turkish family language), the model described above was run using multiple-group linear regression analysis.

We accounted for the nested data (students nested in classes) using the TYPE = COMPLEX command correcting for underestimated standard errors of our model parameters. The regression models for German family language and Turkish family language were estimated simultaneously using the GROUPING command in Mplus. There were no missing values. In case of significant interactions, simple slope tests were performed to determine whether one or both slopes differed significantly from 0 (Aiken & West, 1991), and were visualized using the web-based tool interActive (McCabe et al., 2018).

## Results

### Preliminary results and descriptive statistics

The average completion time for all students was *M* = 47.41 min (*SD* = 10.39). On average, the students knew 5.16 words (*SD* = 2.80) in the pretest and 9.80 words (*SD* = 4.00) in the posttest. This resulted in an average learning gain of 4.64 words (*SD* = 2.95). Regardless of the experimental conditions, students with German family language (*M* = 5.11, *SD* = 2.49) learned more words than students with Turkish family language (*M* = 4.16, *SD* = 3.29), β = −.16 *p* = .091, *d* = 0.16. Table 1 presents the descriptive statistics of vocabulary learning gains for the different experimental conditions, separated by group, as well as the individual comparisons between the conditions.

**Table 1:** *Means and Standard Deviations of the Vocabulary Learning Gains as well as Mean Differences between Experimental Conditions*

Experimental condition	Students with German family language					Students with Turkish family language			
Teacher´s origin/ stereotype activation	Mean A (*SD* A)	Mean B (*SD* B)	β	*p*		Mean A (*SD* A)	Mean B (*SD* B)	β	*p*
Turkish/ Stereotype (A) vs. Turkish/ No stereotype (B)	5.00 (2.72)	4.39 (2.19)	.12	.571		6.64 (2.90)	3.52 (3.11)	.44	.003**
Turkish/ Stereotype (A) vs. German/ Stereotype (B)	5.00 (2.72)	5.32 (2.45)	-.06	.691		6.64 (2.90)	3.79 (3.26)	.40	.004**
Turkish/ Stereotype (A) vs. German/ No stereotype (B)	5.00 (2.72)	6.25 (2.45)	.23	.368		6.64 (2.79)	3.86 (3.12)	-.43	.004**
Turkish/ No stereotype (A) vs. German/ Stereotype (B)	4.39 (2.19)	5.32 (2.45)	.19	.155		3.52 (3.11)	3.79 (3.26)	.04	.733
German/ Stereotype (A) vs. German/ No stereotype (B)	5.32 (2.45)	6.25 (2.45)	-.16	.336		3.79 (3.26)	3.86 (3.12)	-.01	.904
Turkish/ No stereotype (A) vs. German/ No stereotype (B)	4.39 (2.19)	6.25 (2.45)	-.35	.015*		3.52 (3.11)	3.86 (3.12)	-.06	.713

*Notes. N* = 182. All values were estimated using Mplus. * *p* ≤ .05, ** *p* ≤ .01, *** *p* ≤ .001.

### Multiple-group regression

Since an intraclass correlation coefficient (ICC) of 0.01 is already expected to cause an inflation of the alpha error probability, which can lead to biased estimates of the standard errors (Cohen et al., 2003), we accounted for the multilevel structure at ICCs of 0.09 (students with German family language) and 0.08 (students with Turkish family language). Table 2 shows the standardized and unstandardized regression coefficients of the multiple-group regression analysis.

**Table 2:** *Multiple-Group Regression Analysis Predicting Vocabulary Gains*

	Students with German family language					Students with Turkish family language				
	*B*	*SE*	β	*p*		*B*	*SE*	β	*p*	
Intercept	6.53	0.71	2.62	< .001***		4.35	0.75	1.32	< .001***	
Teacher origin^a^	-2.02	0.85	-.40	.018*		-0.49	0.88	-.07	.582	
Stereotype activation^b^	-1.04	0.97	-.21	.302		-0.21	0.68	-.03	.759	
Teacher origin X stereotype activation	1.78	0.95	.25	.064		3.29	1.29	.36	.010**	
School type^c^	-1.65	0.68	-.21	.029*		-0.71	0.73	-.11	.329	

*Notes. N* = 182. All values were estimated using Mplus. Standard errors were adjusted for the nesting of students within classes. ^a^ 0 = German-origin teacher, 1 = Turkish-origin teacher ^b^ 0 = no stereotype activation, 1 = stereotype activation. ^c^ 0 = upper academic track, 1 = lower academic track. *R*^2^_German − origin students_ = .10, *R*^2^_Turkish − origin students_ = .12, ICC _German−origin students_ = .09, ICC _Turkish−origin students_ = .08. * *p* ≤ .05, ** *p* ≤ .01, *** *p* ≤ .001.

Our first research hypothesis was that – as compared to the no stereotype activation condition – Turkish-origin students would be impaired in their learning gains when the negative stereotype about their group was voiced by a German-origin teacher, but not when voiced by a Turkish-origin teacher. As shown in Table 2, the interaction between teacher origin and stereotype activation was found to be significant (β = .36, *p* = .010, *d* = 0.38). However, the pattern of this interaction deviated from our expectation. Students with Turkish family language had the highest learning gains when they were taught by a Turkish-origin teacher who named the stereotype; post-hoc simple slopes analyses confirmed this finding (*B* = 3.09, *SE* = 1.03, *p* = .003). No other predictor than the interaction between teacher origin and stereotype activation was significant for students with Turkish family language. Our model explained a total of 12% of the variance in Turkish-origin students’ learning gains’.

**Figure 2:**
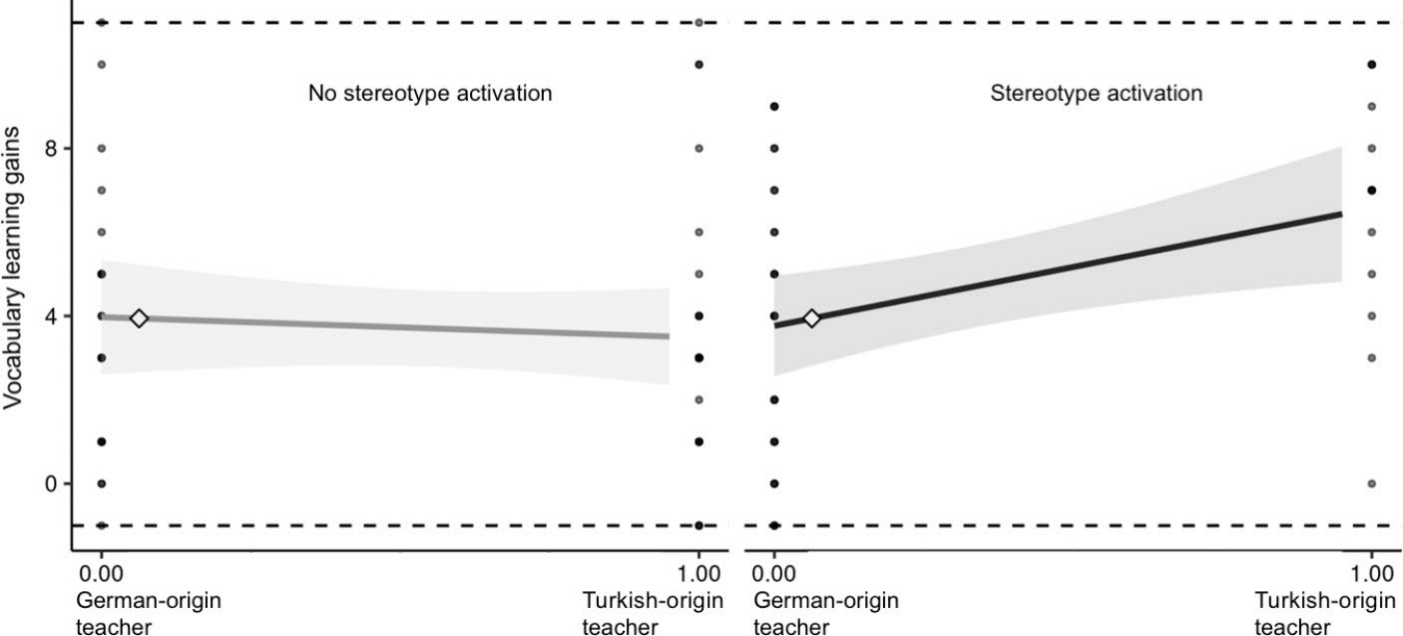
*Simple Slopes Graphs for Students with Turkish Family Language* *Notes*. Simple slopes graphs depicting the relationship between teacher origin and vocabulary learning gains. Simple slopes are displayed for no stereotype activation (left) and stereotype activation (right). School type was entered as covariate. Shaded areas show the 95% confidence region. Figures were created using an application by McCabe et al. (2018).

Our second research hypothesis was that for students with German family language the activation of the negative competence-related stereotype targeting Turkish-origin students would increase their learning gains. The main effect of stereotype activation was, however, non-significant (β = − .21, *p* = .302, *d* = 0.22), indicating that students with German family language were unaffected by the activation of the negative stereotype about students with Turkish family language. Origin of the teacher was a negative and significant predictor of vocabulary gains for students with German family language (β = − .40, *p* = .018, *d* = 0.42), suggesting that they learned more with a German-origin teacher than with a Turkish-origin teacher. For German-origin students, our model explained a total of 10% of the variance in their learning gains.

Regarding the control variable, school type was linked to vocabulary gains in the group of students with German family language (β = − .21, *p* = .029, *d* = 0.22), indicating that students attending schools of higher academic tracks learned significantly more vocabulary than students attending schools of lower academic tracks. No effect of school type was observed for students with Turkish family language.

## Discussion

In an experimental study, we investigated whether a negative stereotype about lower language learning skills in students of Turkish descent impairs their acquisition of new German vocabulary and whether this disadvantage can be alleviated by a teacher of Turkish origin serving as an ingroup model thereby invalidating the negative stereotype. We regressed students’ learning gains on our experimental variables and included school type as a covariate. While German-origin students learned more when attending a “Gymnasium” than an “Integrierte Sekundarschule”, academic track did not play a role in the learning gains of Turkish-origin students. This can possibly be explained by the fact that given equal competencies (as measured via standardized tests) Turkish-origin students are more likely on the higher academic track than their German-origin peers, due to immigrants’ stronger educational aspirations and more optimistic educational decisions (e.g., Lenz et al., 2021; for an overview see Dollmann, 2021), so that learning gains in our experimental vocabulary task differed less between school tracks in the case of Turkish-origin students than in the case of German-origin students.

### Effects of stereotype threat and teacher origin on students of Turkish descent

We had expected that – compared to the control condition – Turkish-origin students would show lower learning gains when a negative stereotype about their group was activated by a German-origin teacher, but not when the stereotype was voiced by an ingroup expert model: a teacher of Turkish descent. Unexpectedly, students with Turkish family language whose teacher was an ingroup member and who were exposed to the negative stereotype about their group improved their vocabulary knowledge at a higher rate than Turkish-origin students in all other experimental conditions.

Why is it that Turkish-origin students (a) did not suffer from the stereotype threat but (b) even profited from it when instructed by an ingroup expert? First, we discuss possible reasons why no negative effect of stereotype threat appeared. This could possibly be due to the fact that the negative stereotype about Turkish students was activated in a learning situation rather than in a performance situation (cf. Rydell et al., 2010). Our experimental situation thus did not correspond to a high-stakes testing setting, which may have attenuated the threat experience in Turkish-origin students (Shewach et al., 2019). If a negative stereotype is activated in a testing situation, individuals’ motivation to disconfirm their group’s negative depiction does not necessarily enable them to perform better as the skills to be demonstrated must have been previously acquired (cf. Latsch & Hannover, 2014; Seibt & Förster, 2004). In contrast, if the stereotype is activated in a learning situation, individuals may experience stronger control being able to disprove the stereotype through increased effort, with stronger engagement in the learning task leading to stronger learning gains. However, this interpretation is not supported by the results of previous studies, which found negative effects of stereotype threat in learning situations as well (Appel et al., 2011; Boucher et al., 2012; Rydell et al., 2010a, 2010b; Taylor & Walton, 2011).

An alternative explanation why Turkish-origin students did not suffer from the stereotype threat is that by referring to a modifiable attribute, i.e., family language, we triggered a weaker threat experience for the stigmatized group than if we had referred to a stable attribute, i.e., ethnic background: negative stereotypes are particularly threatening if they imply that one’s capabilities are immutably and permanently restricted by one’s group membership (Good et al., 2003; Froehlich et al., 2016; Mendoza-Denton et al., 2008). The threat for the Turkish group may have been further weakened by the fact that the stereotype was expressed in relation to families in which Turkish is *also* spoken (i.e., two languages instead of only one), thus implicitly even referring to a greater language competence of this group (see, e.g., Berthele, 2012; Park-Johnson, 2020, for how manifestations of bilingualism are perceived). Finally, it is conceivable that as we activated the stereotype by a blatant cue (Nguyen & Ryan, 2008), i.e., by explicitly referring to Turkish students’ inferior learning capabilities, and removed the threat in the control-condition by a subtle cue (Nguyen & Ryan, 2008), i.e., by referring to potential difficulties of adolescents from *both* family language groups, the two experimental conditions were not sufficiently different from each other in their effect: in the control condition, in principle, each participant could interpret the statement that young people often have difficulties learning vocabulary as referring to themselves. Thus, it cannot be ruled out that due to the negative stereotype about their group, students of Turkish origin were particularly likely to feel that they themselves were being addressed when the teacher spoke of students (in general) having difficulty learning new words and thus felt threatened even though they had been assigned to the control condition.

We now discuss possible reasons why Turkish-origin students even profited from the stereotype activation when instructed by an ingroup expert model. It seems students were particularly motivated to learn with the teacher who combined the following characteristics: 1) she represented their minority ingroup targeted by negative stereotypes, 2) she was a successful ingroup model in her professional role as a teacher, and 3) she openly spoke about potential difficulties students of the stigmatized ingroup may encounter which may seem particularly authentic as she was talking about students who belong to her own group. It is conceivable that by mentioning potential difficulties of Turkish-origin students, the preconditions described by Dasgupta (2011) as well as Marx and Roman (2002) for a successful individual to become a personal role model were met: after all, one core responsibility of teachers is the identification of difficulties that may arise depending on individual learning conditions (here the student’s language background). Perhaps this is why Turkish-origin students in the experimental threat condition were particularly strongly inclined to think of the Turkish-origin teacher as an expert role model, to see her personal success as attainable, and to identify with her (cf. Chaney et al., 2018; Dasgupta, 2011; Marx & Roman, 2002). This interpretation is in line with the view of Marx and Ko (2012) who stress that the ingroup expert model can be particularly effective not because it eradicates the stereotype as such, but because it can mitigate negative consequences of stereotype activation: “We define role models as those individuals who excel in the relevant stereotyped domain. What is particularly appealing about this strategy is that it does not rely on eliminating the threat of stereotype, something that is usually not feasible in real-world situations. Instead, exposure to such role models reduces the negative effects of the stereotype without needing to eliminate the ‘threat in the air’ (Steele, 1997)” (Marx & Ko, 2012, p. 807).

Also, it is conceivable that Turkish-origin students interpreted the teacher’s comment on learning difficulties of students of their ingroup as a message that these difficulties are temporary and surmountable because the teacher had successfully overcome such difficulties herself. This interpretation is supported by the study of Good et al. (2003) who found that stigmatized students improved their test scores when encouraged to view intelligence as malleable or to attribute difficulties to the learning context being new to them. Perhaps the Turkish-origin teacher in our study sent the implicit message that Turkish-origin students should dismiss the negative stereotype about their group because they can overcome their academic difficulties, just as she had done to eventually become a teacher.

### Effects of stereotype threat and teacher origin on students of German descent

For German-origin students, we had expected that the stereotype’s activation would strengthen their learning gains due to ingroup-favorable social comparisons with the derogated outgroup (cf. Walton & Cohen, 2003). Results showed, however, that students with German family language were unaffected by the stereotype activation manipulation. Further, our findings show that they learned more vocabulary with a German-origin teacher than with a Turkish-origin teacher.

The absence of a stereotype-lift effect can be attributed to the same reason already discussed as a possible cause of the lack of a threat effect for the Turkish-origin students: the stereotype threat referred to adolescents who *also* speak Turkish at home, so the German students may have even attributed a higher language competence to their bilingual Turkish peers (see, e.g., Berthele, 2012; Park-Johnson, 2020).

An alternative explanation why no stereotype-lift effect was observed is that German-origin students who participated in our study did not believe in the stereotype about their Turkish-origin peers. While negative stereotypes about people of Turkish descent prevail in Germany (Latsch & Hannover, 2014; Lorenz, 2021; Froehlich & Schulte, 2019), interethnic attitudes between Turkish- and German-origin students need not be negative within the multiethnic classrooms we investigated. Interethnic contact has been found to enhance outgroup-related knowledge and empathy and thus positive interethnic attitudes (Bubritzki et al., 2018; Janmaat, 2012), to the extent that equality and inclusion norms prevail and diversity is considered an important value in the classroom (Schwarzenthal et al., 2018). Hence, it may well be that positive interethnic attitudes dominated in the classrooms we investigated, such that German-origin students did not engage in downward comparisons with their Turkish-origin peers and thus did not profit from stereotype lift. This interpretation is substantiated by a study by Chatard et al. (2008), showing that non-stigmatized individuals profit from stereotype lift only if they endorse the stereotype about the stigmatized outgroup.

That German-origin students learned more with the German-origin teacher is in line with studies showing that a match between student and teacher ethnicity can be beneficial for student outcomes, as teachers can be expected to evaluate and treat students of their ethnic ingroup more advantageously than students of ethnic outgroups (e.g., Egalite et al., 2015; Kleen et al., 2019). The empirical evidence for positive effects of ethnic match is, however, ambiguous (see Driessen, 2015, for a review).

### Limitations

Our study has several limitations. There are many potential moderators of the effects of our experimental treatments which we either did not measure (e.g., students’ perception of the teacher’s competence and expertise, cf. Marx & Roman, 2002) or could not include into our regression analyses (gender) due to our rather small sample size. Moreover, while we investigated stereotype threat in a learning rather than testing environment, our study design does not match the quality of the one in the study by Boucher et al. (2012), in which an additional condition was created where the stereotype threat was removed directly following the learning situation and before the testing situation. Since it was extremely difficult to recruit schools to participate in our study in times of the COVID-19 pandemic, we refrained from implementing such an extended study design. As another limitation, it should be noted that the software LimeSurvey assigns participants to experimental conditions by complete randomness and thus does not ensure equal allocation of participants to experimental conditions. Especially in small samples, this implies a high risk that unequal cell populations result, as has been the case in our study, where cell sizes varied between 27 and 66 participants. Furthermore, deviating from other studies that typically induced stereotype threat by referring to Turkish-origin students’ ethnic background (Froehlich et al., 2016; Martiny et al., 2014; Mok et al., 2017), the threat in our study referred to students’ family language. We have thus compromised a less clear differentiation between students with and without a migration background: our differentiation does not necessarily equate to a distinction based on whether a family immigrated to Germany from Turkey at any point in time. Since the vast majority of adolescents of Turkish descent living in Germany today were born in Germany, asking whether the student themselves were born in Germany or Turkey would not have been a suitable way to distinguish between the two groups of interest to us. According to current data protection laws on the part of the approving school authority in the federal state of Germany where our study was conducted, it is prohibited to ask students whether their parents and/or grandparents were born in Germany (due to the right to informational self-determination on behalf of the parents and grandparents). We therefore cannot rule out that the group of participants we classified as Germans actually included participants whose families have a Turkish background.

Another restriction is that reliability of our word learning measure was only satisfactory in the posttest. In our pilot study, we had selected the vocabulary items based on them being known to as few students as possible, such that they could make correspondingly large learning gains with them. This approach did not necessarily ensure that all vocabulary items were indicators of students’ German language ability because if a word was not known, they had to randomly select their answers in the multiple-choice format. The fact that the reliability of the posttest was then significantly improved compared to the pretest suggests, however, that the ability to learn new German vocabulary was reliably recorded.

Finally, concerning our dependent variable, we wanted to focus on learning gains and how learning gains were affected by the activation of a stereotype. Through intensive pretesting, we identified words for the pretest that were known by very few students, so there was ample room for all students to improve their vocabulary knowledge through the learning task. Still, we cannot ascertain whether there could be differentiating effects of stereotype activation for students with low or high prior vocabulary knowledge.

### Scientific contribution of our study

While our findings deviate from the predicted pattern, they do show that a Turkish-origin teacher helped students from Turkish speaking families to perform particularly well. This result is consistent with research on the impact of ingroup experts on stigmatized individuals, according to which such successful role models can alleviate self-doubt in learning and performance situations (e.g., Chaney et al., 2018; Dasgupta, 2011; Steele et al., 2002; Stout et al., 2011). Going beyond the findings from this line of research, we observed that stigmatized students even profited from the ingroup expert model voicing the stereotype about their common ingroup: the Turkish-origin teacher explicitly addressing the challenges Turkish-origin students have to master helped stigmatized students increase their vocabulary knowledge to a particularly strong extent. It seems, when the stereotype is named by an ingroup expert in a learning situation rather than a testing situation, stigmatized students may feel particularly strongly motivated to counteract the stereotype by effort expenditure and to thus also imitate the ingroup model.

To our knowledge, our study is the first to investigate the effect of an ingroup expert model on students from a stigmatized immigrant group attending school in Germany. This assessment is supported by the findings of the meta-analysis of stereotype threat interventions by Liu et al. (2021): only three of the 251 investigated effect sizes referred to the provision of an ingroup role model sharing stigmatized individuals’ race/ethnicity as the intervention strategy, with all three studies examining whether the negative effects of stereotype threat on the performance of African Americans on a verbal ability test could be mitigated by an expert Black ingroup model. Since, in our study, the teacher who was supposedly from Turkey was portrayed by the same actress as the teacher who was from Germany, it can also be ruled out that the effects were due to other characteristics of the model’s personality.

While the Federal Government of Germany and the Kultusminister (Minsters of Culture) of the federal states in Germany committed themselves to increase the proportion of teachers with a migration background already back in the year 2007 (Die Bundesregierung, 2007), almost 15 years later, with 13% the proportion of such teachers has remained very low (Statistisches Bundesamt, 2021a). Our study results give reason to hope that increasing the proportion of teachers with an immigrant background will contribute to closing the gap in educational attainments between immigrant students and their peers from native families.

## Footnotes

1 Unfortunately, there are no statistics on how many of the teachers with an immigrant background in Germany are of Turkish descent. It can be assumed, however, that they represent only a small proportion of the teachers with a migration background since only 12.6 % of the people with a migration background in Germany are of Turkish origin (Statistisches Bundesamt, 2022, Table 2).

2 As students who had a family language other than Turkish or German were given a different experimental stereotype threat treatment (in which the teacher said students with non-German family language (rather than Turkish family language) would have difficulty learning vocabulary), we had asked teachers about students’ family language before conducting the experiment. In 14 cases, the teachers had indicated that the students would speak Turkish at home, while the students themselves reported a language other than Turkish or German as their family language at the end of the experiment. These students were therefore excluded from further data analysis.
